# Medicines use reviews: a potential resource or lost opportunity for general practice?

**DOI:** 10.1186/1471-2296-14-57

**Published:** 2013-05-06

**Authors:** Asam Latif, Kristian Pollock, Helen F Boardman

**Affiliations:** 1 Division of Social Research in Medicines and Health, School of Pharmacy, University of Nottingham, East Drive, University Park, Nottingham, NG7 2RD, UK; 2 School of Nursing, Midwifery, and Physiotherapy, University of Nottingham, Nottingham, NG7 2HA, UK

**Keywords:** Adherence, Community pharmacy, Cooperative behaviour, General practitioners, Medicines Use Reviews, Patients, Pharmacists

## Abstract

**Background:**

Patient non-adherence to medicines represents a significant waste of health resource and lost opportunity for health gain. Medicine management services are a key health policy strategy to encourage patients to take medicines as they are prescribed. One such service is the English Medicines Use Review (MUR) which is an NHS-funded community pharmacy service involving a patient-pharmacist consultation aiming to improve patients’ knowledge of medicines and their use. To date the evidence for MURs to improve patient health outcomes is equivocal and GPs are reported to be sceptical about the value of the service. This paper presents the patient’s perspective of the MUR service and focuses on the importance of GP-pharmacist collaboration for patient care. Suggestions on how MURs may have value to GPs through the delivery of increased patient benefit are discussed.

**Method:**

A qualitative study involving ten weeks of ethnographic observations in two English community pharmacies. Observations were made of all pharmacy activities including patient-pharmacist MUR consultations. Subsequent interviews with these patients were conducted to explore their experience of the service. Interviews with the pharmacy staff were conducted after the period of observations. A thematic approach was used to analyse the data.

**Results:**

Fifty-four patients agreed to have their MUR observed of which thirty-four were interviewed. Seventeen pharmacy staff were also interviewed. Patients reported positive views about MURs. However, there was little evidence suggesting that pharmacists and GPs were working collaboratively or communicating outcomes resulting from MURs. MURs were conducted in isolation from other aspects of patient care. Patients considered GPs to have authority over medicines making a few wary that MURs had the potential to cause tensions between these professionals and possibly adversely affect their own relationship with their doctor.

**Conclusions:**

This study reveals the potential for effective GP-pharmacist collaboration to improve the capacity of the MUR service to support patient medicine taking. Closer collaboration between GPs and pharmacists could potentially improve patients’ use of medicines and associated health care outcomes. The current lack of such collaboration constitutes a missed opportunity for pharmacists and GPs to work together with patients to improve effective prescribing and optimise patient use of medicines.

## Background

Multiple medicines are often prescribed to manage co-morbidities associated with an aging population resulting in patients having to manage complex medication regimes. A considerable body of evidence suggests that patients have practical problems with taking medicines or may even be reluctant to take them because of concerns about side effects, the consequences of dependency or being unclear about the benefits [1,2]. Patient adherence to prescribed medicines can be as low as 50 per cent and represents a lost opportunity for improving health outcomes with consequent increase in hospitalisation [3-5]. One evaluation estimates that in England the gross annual cost of NHS primary care prescription medicines wastage is £300 million per year including £90 million worth of unused prescription medicines that are retained in individuals’ homes and £110 million returned to community pharmacies [6]. Despite the rising cost of treatment, prescribers may be unaware of the level of patient medicine adherence and may not have time, or be averse to discussing this during the medical consultation [2,7,8]. There is growing acceptance that prescribing is a partnership with patients [9]. However, some patients may not feel the General Practitioner (GP) has time to discuss their concerns [10,11]. There may still be continuing conformity to the traditional patient role and deference to professional authority that limits discussions about treatment [12,13]. There is great scope for interventions to optimise medicines use including improvements in physician communication during prescribing and the promotion of patient-centred care [14-16]. Alongside these approaches are medicine management services such as medication reviews [3,17,18]. In the UK, and internationally, medication reviews are increasingly being formalised and commissioned through community pharmacies as they are seen to support patient medicine adherence [19-23]. These services are part of a range of extended pharmacy services that aim to raise the professional status of pharmacy and make better use of the pharmacist’s skills [24]. However, pharmacists may require consultation skills training for these newer roles to ensure patients’ complex needs surrounding medicines are met [1,2].

This paper is concerned with the English community pharmacy Medicines Use Review (MUR) service which became available since 2005. An MUR involves a patient-pharmacist consultation to discuss the patient’s use of medicines and improve their knowledge about their purpose [20]. Patients are eligible for the service if they have been prescribed two or more medicines and are regular users of the pharmacy. Pharmacists may communicate outcomes resulting from MURs to the patient’s GP by sending them a report of the recommendations [25]. MURs performed with patients with asthma have suggested the most benefit [26,27]. One study aiming to quantify the effects of performing an MUR on GP prescribing for patients with CHD, found 56% of the pharmacists’ recommendations had been actioned [28]. Nevertheless, GPs have expressed concerns that MURs are conducted in isolation from them, that they duplicate work and that paperwork is overcomplicated and unavailable in an electronic format [29,30]. Furthermore, GPs consider MURs to provide little benefit to either their patients or themselves and make no contribution to their contractual Quality and Outcomes Framework (QOF) measures [29-32]. Some GPs may consider the community pharmacist role primarily as a ‘shopkeeper’ and distant from direct patient care [33]. Despite this, over 8,000 English community pharmacies are engaging with, and claiming payment for, the MUR service and in 2011-2012 over 2 million MURs were conducted at a cost of £68m [34].

Researching the MUR service, its implementation and the patient perspective is challenging. There has been little research attempting to explore the MUR consultation as it is naturally implemented in practice as the service is often offered ‘on the spot’ when the patient presents to fill their prescription. The aim of this study was to use a small number of case study sites to develop detailed knowledge of the pharmacy’s implementation of MURs and patient experience rather than more superficial knowledge involving a larger number of pharmacy sites. As such, we make no claims about statistical representation or generalisation to a larger population. A qualitative approach was undertaken which was exploratory, inductive in nature and oriented to answering ‘why’ and ‘how’ questions. By investigating MURs in this way, we can learn about how people respond in their natural settings and so achieve a better understanding of their perception of MURs and how the service fits into the system of wider health care. The aim of this study was to investigate the patient perspective of the MUR service and this paper examines the scope for more effective GP-pharmacist collaboration in order to develop the MUR as resource to improve health outcomes for patients.

## Methods

### Recruitment of pharmacy sites

Following approval from the East Midlands (Nottingham 2) NHS Research Ethics Committee, two English community pharmacies, one ‘multiple’ (part of a chain retailer) and one ‘independent’ (defined in the UK as up to five pharmacies with the same owner) were recruited to learn about the different contexts in which MURs were being performed. A pragmatic approach was taken to recruit the multiple involving contacting AL’s previous employer and seeking permission to perform the research in one of their branches. Permission was sought from the Company’s Head Office and a pharmacy was selected based upon the pharmacy conducting a reasonable number of MURs to ensure recruitment to the study. Pharmacies that the researcher had previously worked in regularly were avoided to reduce the potential of being mistaken, by pharmacy staff, for the pharmacist on duty.

The independent pharmacy was identified from a list of all the independent pharmacies that were located in the Nottingham and Nottingham County PCT areas. Pharmacies were identified by a member of the University’s pharmacy academic team who was involved with undergraduate community pharmacy placements and through a local locum pharmacist who had local knowledge of which pharmacies were actively offering the MUR service. Five invitation letters were sent during August 2008 inviting the pharmacy to the study. However, all reported either not regularly performing MURs on a regular basis or reported being ‘too busy’ to participate. Another five independent pharmacies were identified and approached. This time, only one pharmacy reported regularly performing MURs and expressed interest in taking part in the study.

### Fieldwork

Consent was obtained from the pharmacists and support-staff for five weeks of observations in each pharmacy. One-week placements over a 12-month period between November 2008 and October 2009 allowed the data collection and analysis phases to proceed simultaneously. Ethnographically-oriented unstructured observations were made by AL of all pharmacy activities that seemed relevant to the situation being studied and the context in which they occurred, including all activities relating to the MUR service. Pharmacies were requested to display posters within the pharmacy to promote patient awareness of the study. All pharmacy staff were requested to identify and invite patients for MURs as per normal practice and introduce the research to those who accepted the offer of an MUR. Eight patients were observed to decline the offer of an MUR: one patient from the independent and seven from the multiple. All the remaining patients who were invited agreed to an MUR and to participate in the research; at this point AL was introduced. MURs were carried out within a private consultation room and AL made hand written notes of the patient-pharmacist interaction with a fuller account being written up afterwards. Audio or video recording the MUR consultation would have provided verbatim data. However it was decided, upon considering the ethical issue of recording patient’s MUR consultation with little prior notice, that hand written notes would be used. This would produce a less detailed account of the MUR but it was felt that this was less intrusive and a necessary compromise.

In order to build on the researcher’s account and to triangulate the findings, interviews were held with both patients and pharmacy staff. Patients were invited to take part in a semi-structured interview about the experience to clarify, confirm and expand on the observational data. After the pharmacy observations were completed, pharmacy staff took part in interviews to discuss their perceptions of the MUR service. Details of the interview topic guides have been reported elsewhere [35]. Permission was obtained from participants for direct quotes to be used in reports and publications. In this paper pseudonyms have been used in quoted extracts to maintain respondents’ anonymity.

Data analysis was iterative and started during the early stages of data collection. All observation field note documents were typed up, interviews transcribed verbatim and the data then imported into the qualitative software programme N-Vivo8. A thematic approach to analysing the qualitative data was used and involved initially reading and re-reading each section of the text and collating them under different headings or ‘codes’ [33,36]. Codes were inductively constructed based upon what was observed and reported in interviews and then systematically read through and the contents condensed, synthesised and narrated. The principle of constant comparison was used to develop and refine generated themes [36,37] which allowed examination of how MURs were constructed, interpreted and contextualised within the overall management of the patient’s health care.

## Results

### Setting and participants

The multiple pharmacy was located in a relatively affluent town, on a busy high street and the independent in a similarly affluent but residential suburb. The number of prescription items that was dispensed from each pharmacy was approximately the same (1600–1700 per week). Fifty-four patients consented for AL to observe their MUR consultation of which 34 patients agreed to be interviewed about their experience of the MUR. Patient interviews were typically conducted a week after the observed MUR and took place at the pharmacy (two at the University of Nottingham), lasted approximately 45 minutes and were audio-recorded. After the observations all five pharmacists (two employees from the multiple; one owner, one employee and one regular locum from the independent) who had been observed during the study were interviewed plus 12 (out of 14) pharmacy support-staff.

### The MUR

Invitations to take part in MURs were typically initiated by the pharmacy staff in an *ad hoc* way when patients attended to collect prescriptions. Both pharmacies had previously tried making appointments with patients but these were seen to be problematic when patients did not attend. Particularly in the independent, patients with whom the staff appeared to have a good relationship were typically selected for an MUR. Although GPs *can* refer patients, no MURs were initiated via this route. MURs were not understood by patients to be a collaborative activity involving or relevant to, the GP but as a quick pharmacy oriented activity to “*check*” their medicines:

Primrose: The lady came up to me and said would I mind going through my medication with the pharmacist and just to kind of make sure that we both knew why this medication was being prescribed and it was just something that chemists are having to do now.

Patient 56yr. F

Among most patients involved in this study the pharmacist did not identify many, or even any, problems with their medicines. Most patients reported the consultation had not improved upon their knowledge of their medicines and rarely affected their use. They were generally satisfied with the current level of knowledge they had about their medicines, being familiar with drugs prescribed for long-term conditions:

Jill: [Sighs] Well I don’t think I’ve got no more knowledge. I think it’s just that I’ve been on these for so long and once you’ve been on them for so long, the doctor does make sure that you’re alright with them.

Patient 64yr. F

Despite a lack of evidence that the MUR service was achieving its intended policy aims, nearly all patients spoke of the MUR positively, describing the review as “*satisfying*” or “*interesting*”. All patients reported feeling comfortable speaking with the pharmacist who they saw as a knowledgeable expert on medicines. Furthermore, they valued the time the pharmacist spent with them, commenting that this had made them feel special, and appreciated the opportunity to speak to them privately. Most accounts suggested that the pharmacist had reassured patients about their medicines:

Researcher: To what extent then did you personally find the review useful?

Esther: Well, I think it gave me a bit of confidence that the pharmacist was caring enough to go though all my medication to make sure I was happy with it…and that I knew what I was doing…

Patient 61yr. F

Comfort: I think it gives you more confidence, it does me, gives me more confidence to think I’m doing the right thing and taking the right medicine

Patient 72yr. F

Although pharmacy staff tended to invite patients with less complicated medicine regimes, there were a few instances during the MUR where the pharmacist identified particular concerns about the patient’s health or medicine. In these cases, rather than intervene directly and on the patient’s behalf, pharmacists preferred to place the onus on patients to return to their GP for resolution of these issues. In the following example the pharmacist identifies a potential deterioration in the patients’ asthma control. Whilst this event may not have been highlighted unless the pharmacist had initiated the MUR, the limited remit and lack of collaboration with the GP led to the pharmacist closing off discussion and failing to ensure that the issue was resolved:

Jane [Pharmacist]: The Ventolin…

Mia [Patient]: Which ones that? [The patient looks in her bag and takes out a Ventolin inhaler].

Jane: That’s the one, how often do you use that?

Mia: It depends, but I'm using it a lot.

Jane: Are you using it eight puffs or more?

Mia: More than that.

*Jane: You should go to see the asthma nurse or doctor because the others are not doing their job. If it’s been a few days you need to see them*^*a*^*.*

MUR 32

The follow-up interview with the patient revealed that the pharmacist’s advice appeared to have been accepted although it is not known whether the suggestion was subsequently followed through:

Researcher: … Did you pick up anything that you didn’t already know?

Mia: …Only that uh I needed to go back, ‘cause the Ventolin. I just thought it was me getting worse… I thought I was on the most I could go on, you know and I’d have to tolerate it. But with her saying that, she said that they can help you more.

Patient 66yr. F

In another case, having discovered non-adherence to a diuretic tablet, the pharmacist encouraged the patient to take the medicine as prescribed. A shared decision was made about informing the GP which was done via the MUR documentation:

The conversation turns to the patient’s furosemide…

Moya [Patient]: I don’t have ankle swelling so I don’t take them [furosemide] every day.

Rebecca [Pharmacist]: You need to take them every day as if you don’t then the kidneys have to work more.

Moya: I will mention that to him [the GP], I've got to see him.

Rebecca: I’ll put that on the [MUR] form [Rebecca explains that the patient should be taking the tablet daily as the fluid builds up and “it’s harder for the kidneys to work to clear the fluid”].

Rebecca: Is it ok for me to let the GP know?

Moya: Yes, I’m seeing him next week.

MUR 9

In the follow-up interview with the patient it was found that without the MUR, the patient would not have brought up the matter with the GP. The patient’s awareness that the pharmacist would be informing her GP through the MUR form both legitimised and encouraged her to raise the matter with the GP:

Moya: … You see my frusemide …I thought well I don’t get any swelling of my ankles so do I need it every day? So, I get a bit naughty and I don’t take them every day. And so of course the pharmacist got on to me and so I’ve got to tell the doctor…whether it is something I should be taking every day…

Researcher: OK, would you have discussed it with the doctor if the pharmacist hadn’t mentioned it?

Moya: No, no I’d probably wouldn't …I might have thought about it and thought well better not say anything else because I might not be doing the right thing [laughs]… but I will mention it. I’ll have to because that form’s gone to him [laughter].

Patient 79yr. F

Another important finding that emerged from the data was how some patients anticipated that the MUR might affect their relationship with the GP. A few patients expressed their awareness of a difference in professional status and hierarchy between pharmacists and GPs and the possibility their doctor might be annoyed to find the pharmacist ‘interfering’ with their medicines without this being clearly sanctioned by the prescriber. As a result, some felt wary of the pharmacist’s involvement or felt that they were going behind the doctor’s back, and that MURs had the potential to cause inter-professional tension or conflict:

Ashley: I don’t think they [GP] like it, outside interference…being from a novice, a pharmacist or anybody else…

Patient 67yr. M

Nicola: I just recently started taking paracetamols…I did ask the doctor if I could take up to six and she said eight. So I just wanted to make sure with the other tablets…I wouldn’t want my doctor to know that [laughs]… I didn’t want to upset the doctor by thinking I was asking her if it was OK to take them … But I just wanted to check…

Patient 68yr. F

### The pharmacist perspective

All the pharmacists interviewed reported enjoying the activity of undertaking an MUR as this provided greater personal patient contact and added diversity to their daily routines. However, they also recognised barriers to effective implementation of the service. MURs were being shoehorned alongside existing duties without additional resource. Lack of patient awareness of the availability and potential value of the service made recruiting patients difficult. As a result, pragmatic strategies were employed by pharmacy staff to offer MURs to patients who met the *minimum* inclusion criteria and who were judged likely to respond to an invitation. Patients with many medicines or those who were perceived to have more complex conditions such as mental illness were avoided for fear that the consultation would be too lengthy. Moreover, organisational pressure on pharmacists to avoid financial loss by meeting targets for the number of completed MURs was evident, particularly among those working in the chain pharmacy:

Jane: Well, it’s not ideal because you’re looking at figures rather than the actual quality of the service that you’re giving…

Employee pharmacist, Multiple

Added to these organisational constraints, when pharmacists were asked about the value of MURs as an inter-professional collaborative activity, they reported that patients were not referred from their GPs and they received little or no feedback from them about any recommendations they made regarding medicines which resulted from the MUR. Consequently, MURs were not seen to foster inter-professional collaboration: rather, pharmacists reported the opposite view:

Kate: I don’t think they’re [GP's] keen on us doing it, to be honest, to be truthful.

Employee pharmacist, Multiple

## Discussion

### Summary of the main findings

The MUR service has failed to capitalise on a potential opportunity to foster inter-professional collaboration to support patients to take medicines appropriately. GPs did not refer patients for MURs and there was no evidence to suggest that pharmacists and GPs were working in partnership to identify the patients who could benefit most from the service. Pharmacists lacked adequate resources to implement the service and felt under pressure to reach targets which caused them to adopt pragmatic strategies of patient selection, and to routinize the process of the MUR consultation [38]. Although GPs were notified about concerns or recommendations resulting from the MUR, pharmacists received no feedback, and did not engage in dialogue with GPs about their response to these. Deference to GPs’ professional status and pharmacists’ perception that the MUR service was disregarded by GPs further limited the usefulness of the service. Pharmacists tended to place the onus on patients to take up issues arising from the MUR with their GP, which many may have been reluctant to do, rather than intervene directly. Most patients reported little improvement in their knowledge of medicines or alteration to the way they took them. Nevertheless, targeted effectively, MURs have the potential to reveal important medicines and health related issues with substantial impact on healthcare outcomes. Our findings suggest that there is scope for developing a much more effective and joined-up service that transcends traditional boundaries between GPs and pharmacists, reduces the occurrence of medicine related problems presented to GPs and supports patients to obtain greater benefit from their treatments.

### Comparison with existing literature

Surveys have been used to investigate satisfaction with the MUR service [26-28,39]. However, there has been little research investigating ‘live practice’ of the MUR service and the processes that lead up to and shape the MUR consultation. This study supports previous research indicating that MURs have not significantly contributed to improving GP-pharmacist relationships or collaboration. Pharmacists’ perceptions of GP views about MURs, along with continuing deference and reluctance to question GP prescribing decisions, have limited potential collaborative working opportunities [28-31,40,41]. This is unfortunate as the literature reports positive results on patient health outcomes when effective GP-pharmacist collaboration is promoted [42-45]. Furthermore, this study adds to existing concerns about how corporate pressure for pharmacists to meet targets can adversely affect the recruitment of patients who could benefit most from the MUR service [30,31,41]. Whereas the GP perspective of MURs has been reported by others [29,30,32], further research is needed to pilot and test more integrated collaborative working practice.

### Implications for future research or clinical practice

Recent reforms to the way pharmacists select patients have been introduced and include measures that half of patients invited for MURs are required to be selected from nationally set target patient groups. These include: 1. patients taking ‘high risk’ medicines (e.g. anticoagulants, diuretics), 2. recently discharged patients who have had changes made to their medicines whilst in hospital and 3. those prescribed medicines for respiratory disease [46]. However, with no signposting from GPs some patients may remain wary of the purpose of MURs or be reluctant to fully engage with the service. GPs could, should they choose, help refer their ‘neediest’ patients for additional support and seek feedback from pharmacists so they can better monitor the patient’s condition between visits [47]. GPs could also save themselves time by diverting minor concerns about prescribed medicines to discussion with the pharmacist. Research into collaborative working practice is needed to show how MURs may complement GP practice-based medication reviews [3]. Research is also needed to find the most effective way of educating patients on the extended community pharmacist’s role and MUR so they feel the activity is approved by all involved in their care and is genuinely for their benefit. Community pharmacists will also have to respond and better organise resources required to deliver MURs and work with practice managers locally to build mutual rapport and trust if patients are to capitalise on the potential benefits offered by this service.

Policy makers should consider integrating pharmacy medicines management services such as the MUR and the recently introduced New Medicines Service [48] into the QOF measures to incentivise GP engagement and position MURs so they complement existing GP medication reviews. Such measures, as with any collaborative activity, are dependent upon the willingness of GPs to provide the service mandate and legitimacy and pharmacists’ readiness to change their practice in order to work in partnership [17,43-45]. This should encourage shared values and a common purpose when delivering MURs so that patients can benefit from a more effective and appropriately targeted service.

### Strengths and limitations of the study

To our knowledge this is the only observational study that has explored MURs as they occur naturally within a pharmacy setting. This study used a combination of two qualitative research methodological approaches to enhance the credibility of the findings. The triangulation of direct observation (researcher’s accounts) with accounts provided by respondents in interviews provided a powerful means of understanding the complexity of respondents’ views, how these may shift contextually, the situational pressures which underlie them and the resulting difference in what people ‘say’ and what they ‘do’. These findings make a significant contribution to the understanding of how patients contextualise their experience of the MUR and the significance of the GP-pharmacist relationship.

A well known limitation to fieldwork observations is the unknown effect of the researcher’s presence on participants. The longitudinal nature of the study was intended to reduce the extent to which participants modify behaviour as a result of a heightened awareness of the observer. However, the extent to which this effect may have influenced our results is unknown. Due to the cross-sectional design of the study it is difficult to assess the extent to which advice provided by the pharmacist was followed by patients over the longer-term. Two pharmacies were used as study sites and both shared some characteristics such as levels of affluence in the patient catchment area and volume of prescriptions dispensed. Other pharmacy settings, including ones that may have had more support staff, more diverse patient populations or different relationships with local GP surgeries, could have resulted in pharmacists implementing and performing MURs in a different way and consequently patients perceiving the service differently. Since conducting this study, suggested questions to be asked during the MUR and changes to the format of the MUR have been made [46]. Future research should seek to explore what impact these changes have had on patient care and their perspective of the service

## Conclusions

Medicine management services are a key UK health policy strategy to ensure patients take medicines as prescribed. These services are increasingly being commissioned through community pharmacies to support patients with medicine adherence. Closer collaboration between GPs and pharmacists could potentially improve patients’ use of medicines. The current lack of such collaboration constitutes a missed opportunity for pharmacists and GPs to work together with patients to improve effective prescribing, optimise patient use of medicines and to improve health outcomes.

### Ethical approval

Nottingham NHS Research Ethics Committee.

## Endnote

^a^ The extracts from the observed MURs that are presented in this paper are taken from detailed notes written up after each observation, rather than verbatim quotes.

## Abbreviations

MUR: Medicines use review; QOF: Quality and outcomes framework; GP: General practitioner.

## Competing interests

Dr Latif’s Ph.D. was funded by the Economic and Social Research Council (ESRC) and Medical Research Council (MRC). The ideas expressed in this manuscript are those of the authors and in no way are intended to represent the position of the funding council.

## Authors’ contributions

This study formed part of AL’s PhD. HB and KP were AL’s academic supervisors. All authors contributed to the design of the study and analysis of the data. AL carried out the fieldwork and drafted the manuscript. All authors read and approved the final manuscript.

## Pre-publication history

The pre-publication history for this paper can be accessed here:

http://www.biomedcentral.com/1471-2296/14/57/prepub
